# Arginine as a Synergistic Virucidal Agent

**DOI:** 10.3390/molecules15031408

**Published:** 2010-03-08

**Authors:** Satoshi Ohtake, Tsutomu Arakawa, A. Hajime Koyama

**Affiliations:** 1 Aridis Pharmaceuticals, 5941 Optical Court, San Jose, CA 95138, USA; 2 Alliance Protein Laboratories, 3957 Corte Cancion, Thousand Oaks, CA 91360, USA; 3 Division of Virology, Department of Cellular and Molecular Medicine, Wakayama Medical University Graduate School of Medicine, Wakayama 641-8509, Japan

**Keywords:** virus inactivation, arginine, virus aggregation, infectivity, disinfectant

## Abstract

Development of effective and environmentally friendly disinfectants, or virucidal agents, should help prevent the spread of infectious diseases through human contact with contaminated surfaces. These agents may also be used, if non-toxic to cells and tissues, as chemotherapeutic agents against infectious diseases. We have shown that arginine has a synergistic effect with a variety of virucidal conditions, namely acidic pH and high temperature, on virus inactivation. All of these treatments are effective, however, at the expense of toxicity. The ability of arginine to lower the effective threshold of these parameters may reduce the occurrence of potential toxic side effects. While it is clear that arginine can be safely used, the mechanism of its virus inactivation has not yet been elucidated. Here we examine the damages that viruses suffer from various physical and chemical stresses and their relations to virus inactivation and aggregation. Based on the relationship between the stress-induced structural damages and the infectivity of a virus, we will propose several plausible mechanisms describing the effects of arginine on virus inactivation using the current knowledge of aqueous arginine solution properties.

## 1. Introduction

Acquiring specific immunity through vaccination is the most effective way to prevent viral infections [1,2,3]. Daily preventive measures also protect us from various infections. Such preventive measures are normally accomplished by the application of disinfectants, or virucidal agents, to inactivate viruses on contaminated surfaces or on human fingers and hands. Unfortunately, these reagents can be severely toxic to cells and tissues and hence cannot be used as practical chemo-therapeutic agents [4,5,6,7,8,9]. Thus, development of a safer and effective disinfectant should be a valuable weapon against infectious diseases. We have observed that arginine has a synergistic effect with acidic pH, elevated temperature and other virucidal conditions on virus inactivation [10,11,12,13]. Arginine lowered the threshold of their effective range so that they no longer caused unacceptable side effects. This is based on the observation that aqueous arginine solution inactivated herpes simplex and influenza A viruses [10,11,12] and exhibited antiviral activities on several other enveloped viruses [10,11,12,13]. Due to the reduced safety concerns, aqueous arginine solution may be used not only as a disinfectant, *i.e.*, for hand- and mouth-wash, but also as a virucidal agent at the site of infection in the form of spray or mist. 

Arginine is one of the twenty natural amino acids and, as a normal cell metabolite, is considered to be safe for human use. This amino acid has been found to have very unique properties; *i.e.*, arginine is highly effective in suppressing protein aggregation [14,15,16], increasing the solubility of proteins [17,18], interacting with lipids [19,20], and weakening protein binding in a number of column chromatography methods [21,22,23,24,25,26,27]. Stimulated by such unique nature of arginine, we have been exploring its capability as a virucidal agent. Conventional virus inactivation processes include moderate heating, so called pasteurization, for intravenous immunoglobulin and low pH treatment for recombinant mammalian-derived biopharmaceuticals [28,29]. We have observed a synergistic effect of arginine with heating and low pH to enhance virus inactivation [10,11,12,13] and postulated that such enhanced virus inactivation may be utilized *in vivo* for topical infectious diseases. Here we review both the *in vitro* and *in vivo* virus inactivation effects of arginine, the mechanism of various stresses that cause virus inactivation, and propose a plausible mechanism by which arginine inactivates viruses. 

## 2. Summary of Virucidal Activities of Arginine

### 2.1. *In vitro* virus inactivation

**Figure 1:**
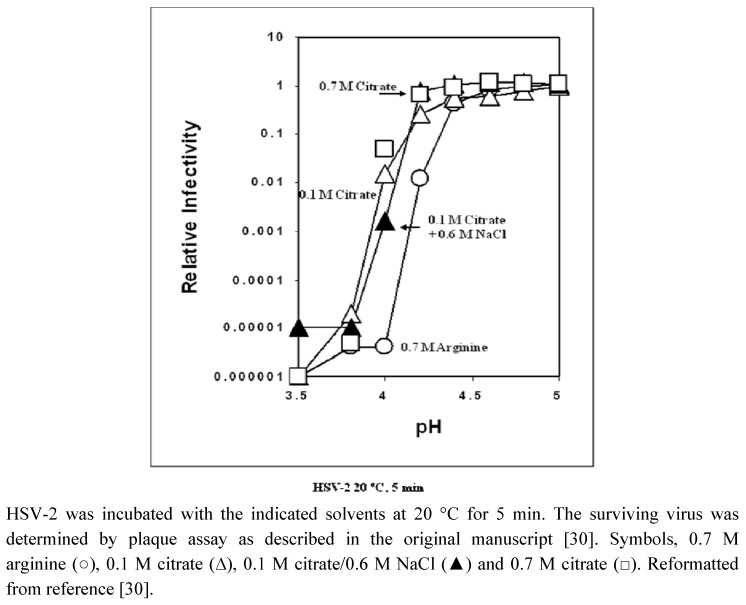
*In vitro *inactivation of HSV-2 by acidic solvents as a function of pH.

*In vitro *virus inactivation effects of aqueous arginine solution were evaluated by incubating the virus preparations with various solvents as a function of pH or temperature. After incubation, the surviving infectious viruses were determined by infecting appropriate host cells with viruses and measuring the plaque formation of the infected cells. With enveloped viruses, arginine has consistently shown a synergistic effect with solution pH and temperature on virus inactivation. Typical inactivation data with an enveloped virus, HSV-2 (herpes simplex virus type-2), are shown in Figure 1 [30]. Similar trend has been observed for other enveloped viruses, including HSV-1 (HSV type-1) and influenza virus [10,11,12,13], and at various incubation temperatures. The results in Figure 1 were obtained following incubation of the virus with the indicated solvents as a function of pH at 20 ºC for 5 min. Virus inactivation was enhanced at a higher temperature and longer incubation time. Acid titration was conducted using 0.1 M citrate and the pH was adjusted by NaOH. As shown in Figure 1, virus inactivation was marginal at pH 4.2 (open triangle), below which a sharp decrease in virus yield was observed. Nearly 5 log_10_ reduction was observed at pH 3.8. Neither the increased citrate concentration at 0.7 M (open square) nor the addition of 0.6 M NaCl (closed triangle) resulted in a shift on the pH titration curve. Although marginal, higher concentration of citrate and NaCl appeared to suppress pH-induced virus inactivation above pH 4.2. 

The above results indicate that neither 0.6 M NaCl nor excess citrate (*i.e.*, 0.6 M) have a synergistic effect with pH (*i.e.*, 0.1 M citrate) on virus inactivation. Conversely, arginine demonstrated synergy with pH in inactivating viruses. Figure 1 shows pH titration of virus inactivation in 0.7 M arginine (arginine HCl solution titrated with HCl). It is evident from the figure that the pH dependence was shifted to a higher pH, by about 0.3–0.4 pH unit. This suggests that to achieve a 5 log_10_ reduction, the solution pH can be maintained at ~4.0–4.1 in the presence of 0.7M arginine, while the same reduction in infectivity can only be achieved at a pH of ~3.8–3.9 in its absence. Although this difference in effective pH range appears to be marginal, the difference could have a significant impact on patient safety if these acidic solutions are utilized in circumstances that require application for human body surface, or perhaps accidental contact. Similar synergistic effects of arginine with a variety of environmental conditions (*i.e.*, pH and temperature) have been observed with several other enveloped viruses [32,33]. 

**Figure 2:**
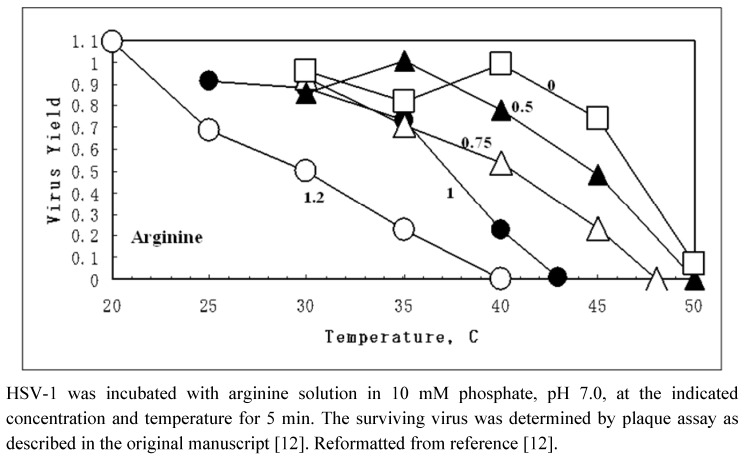
Inactivation of HSV-1 by arginine at neutral pH as a function of temperature.

Arginine at neutral pH also demonstrates synergy with temperature in inactivating viruses [12,13]. Figure 2 shows an example of HSV-1 inactivation with temperature (This sentence was moved here from the top of Figure 2). In phosphate-buffered saline (PBS), HSV-1 was stable up to 40 ºC, above which the infectivity of the virus decreased sharply. There were few surviving viruses at 50 ºC. The temperature dependence of virus inactivation was gradually shifted to lower temperatures with increasing arginine concentration at neutral pH. In the presence of 1.2 M arginine, there was already 50% virus inactivation observed at 30 ºC. The temperatures at which 50 (second column) and 90% virus inactivation (fourth column) occurred are summarized in Table 1. 

**Table 1:** Temperature of 50 and 90% virus inactivation with either aqueous arginine or NaCl solution buffered with 10 mM phosphate, pH 7.0. The concentration of both arginine and NaCl are indicated in the table.

Solvent	Temperature at 50% inactivation		Temperature at 90% inactivation	
0 (PBS)	47		50	
	Arginine	NaCl	Arginine	NaCl
0.5 M	-2	0	-3	
0.75 M	-7	-4	-5	-2
1.0 M	-10	-6	-10	
1.2 M	-17	-7	-16	-4

HSV-1 was incubated for 5 min with arginine solution at pH 7.0 at the indicated concentration. The data are extracted from Figure 2. Inactivation temperature in PBS (phosphate-buffered saline) was subtracted from the temperature in test solvents. Reformatted from reference [12].

Both temperatures decreased with increasing arginine concentration, leading to 16–17 ºC lower temperatures in 1.2 M arginine. Table 1 contains the inactivation data with NaCl. Aqueous NaCl solutions at the indicated concentrations (0.5–1.2 M) also decreased the inactivation temperature, but to a lesser extent. For example, 1.2 M NaCl reduced the temperatures of 50 and 90% inactivation only by 7 (third column) and 4 ºC (fifth column). The results with NaCl suggest that increasing ionic strength is responsible for inactivating the virus. At least a part of virus inactivation by arginine may be due to its ionic property, although virus inactivation by NaCl and arginine may be due to an entirely different mechanism. We will analyze the possible mechanism of arginine effects from the types of damages that viruses suffer and how arginine, or ionic strength, may interrelate to these damages.

### 2.2. *In vivo* virus inactivation

The results described above were obtained in the test tubes. It would be a challenging idea to test the effects of arginine *in vivo*. The time course of inactivation demonstrated that inactivation occurred shortly after the exposure of the virus to arginine solution, suggesting that even a brief exposure of the virus to arginine solution is sufficient. Such a fast action of virus inactivation suggests the potential usefulness of arginine as a non-irritative disinfectant. Conventional disinfectants generally cannot be used on the mucosal membranes or on the injured sites of the body due to their severe cell and tissue toxicities. We anticipated that arginine, being a natural product of lower toxicity, would have insignificant adverse effects on these surfaces upon topical applications and hopefully may exhibit some efficacy. To our surprise, viruses were significantly inactivated by acidic arginine solution on the body surface in two model systems, *i.e.*, HSV-1 infection on epithelial keratinocytes and HSV-2 genital infection [31].

In an epithelial keratinocyte model, HSV-1 was inoculated in the cornea of rabbit eyes and treated with an acidic arginine solution. Those eyes treated with saline (negative control: eyes without treatment) showed severe lesion. In contrast, the eyes treated with a commercially available acycloguanosine ointment demonstrated potent efficacy with no lesion. The application of an acidic arginine solution onto the infected eyes resulted in lesion on the cornea, however to a lesser extent than those treated with saline alone. Citrate of the same acidity did not exhibit such a clear suppression. Although the concentration and pH of the acidic arginine solution were not at physiological conditions, it should be noted that no apparent injuries to the cornea of the treated rabbit’s eyes were observed [31]. The decreased efficacy and toxicity may be in part due to the dilution of acidic arginine solution, upon administration, by the massive liquid secretions. Nevertheless, these results indicate that arginine can suppress the occurrence of infection and the development of keratinocytic symptom by HSV-1 infection in rabbit’s cornea and may warrant further investigation on a more effective formulation and administration route.

We then examined the effects of arginine treatment on the establishment of genital infection with HSV-2 [31]. HSV-2 is also sensitive to the virucidal effects of arginine, significantly more so than HSV-1 [30]. BALB/c mice are sensitive to HSV-2 infection and the virus first multiplies at the site of infection in the vagina, then invades into the central nervous system through the sacral ganglia neuronal cells, and finally causing lethal encephalitis at the brain stem. Mice, if untreated, start dying at 8 days post infection. The infected mice treated with 1.5 M arginine solution (pH 3.5) demonstrated a greatly enhanced survival rate [31]. These results clearly show that the treatment with acidic arginine effectively suppressed the establishment and development of HSV-2 infection in the genital organs of mice, suggesting its potential development as a human therapeutic against genital HSV-2 infection.

## 3. Mechanism of Virus Inactivation by Physical and Chemical Stresses

**Figure 3:**
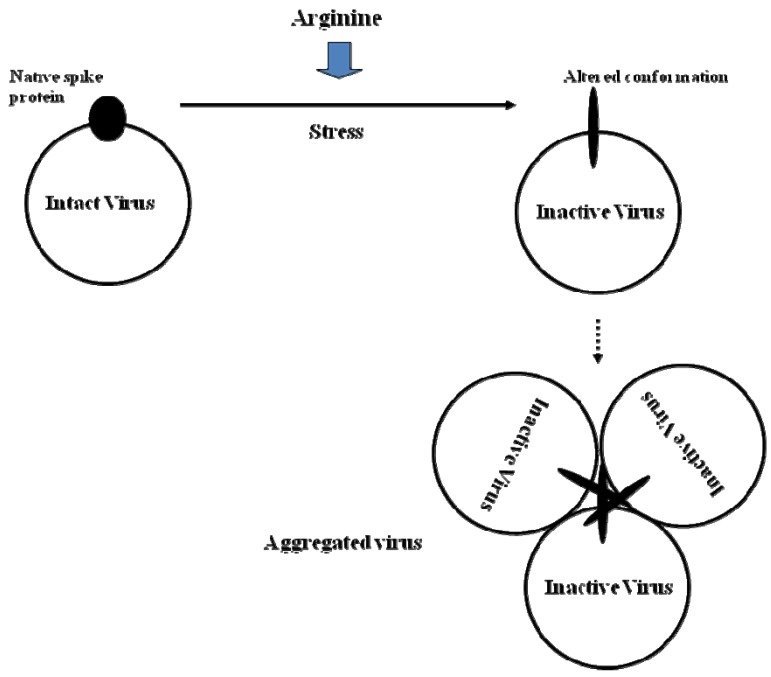
Virus inactivation mechanism-1: synergistic effect of arginine with other stresses causes structural changes in viral spike proteins leading to aggregation.

It is evident that virus inactivation by arginine can only be observed in the presence of other stresses, which may include pH, temperature, freezing, drying, and the presence of antibodies/proteins: so far only pH and temperature were tested. In other words, arginine may synergize with these stresses; in the previous section, the stresses described were heat and acidification. It is important to understand the impact of these stresses on viruses in order to evaluate the synergistic effects of arginine. In general, when both temperature and pH were varied, each virus demonstrated a range of solution pH values in which it was stable and a temperature threshold below which it exhibited structural stability. The most important parameter that is affected by the stresses may be the presence of a viral envelope. Enveloped viruses, such as measles virus and RSV (respiratory syncytial virus), demonstrate increased stability under alkaline conditions [32,33], while non-enveloped viruses, such as adenovirus and Norwalk VLP (virus-like particle), exhibit stability under acidic conditions [34,35,36]. In order to understand how arginine inactivates viruses, we will review the effects of these stresses on the viruses. The main cause of virus inactivation is perturbation of viral surface proteins, as schematically depicted in Figure 3. Low pH alone, or in combination with high temperature, induces conformational changes in the viral proteins, and as a consequence, virus inactivation (Mechanism-1). 

The physical properties of the virus may also have a profound impact on determining its stability, with respect to changes in solution pH and temperature. One critical physical parameter is virus aggregation, as also depicted in Figure 3. Virus aggregation frequently occurs upon application of stress, such as pH shift and increasing temperature. It is thus possible that such aggregation may augment virus inactivation, resulting from changes in viral structure. If the aggregated virus is simply a consequence of inactivation by the stresses [34,35,36], then Mechanism-1 is responsible for virus inactivation. However, if aggregation occurs on viruses that are still infectious, then the effects of stresses may differ from those in the absence of aggregation. 

**Figure 4:**
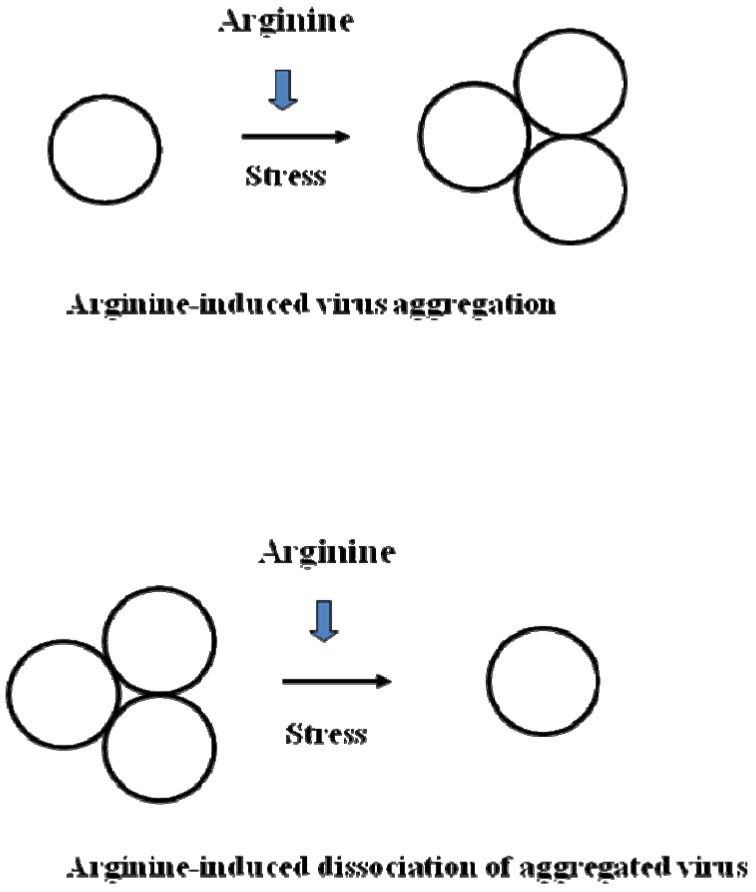
Virus inactivation mechanism-2: arginine in the presence of other stresses causes aggregation or dissociation of virus particles depending on their initial physical state.

Mechanism-2 is related to aggregation of infectious viruses (Figure 4). Specific antibodies have been shown to induce virus aggregation, often without causing structural changes of virion components [37]. Those viruses are still infectious, upon release from the aggregated clumps, and resistant to various stresses commonly causing inactivation. Virus inactivation by these three different mechanisms is summarized below. 

**Figure 5:**
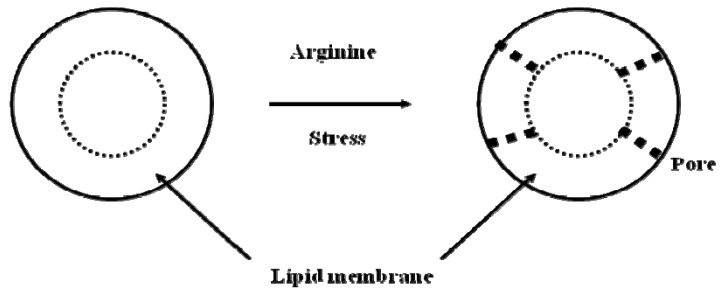
Virus inactivation mechanism-3: synergistic effect of arginine with other stresses causes pore formation in the lipidic viral envelope.

Certain stresses can affect membrane lipids or protein-lipid interface. These stresses may cause conformational changes in proteins, which in turn lead to alteration in membrane properties. Alternatively, they can directly damage membrane lipids, as was the case for defensins, which were shown to induce pore formation in the viral lipids, leading to exposure of viral core genome [38,39]. These observations are summarized in Mechanism-3 (Figure 5).

### 3.1. Mechanism-1

Low pH alone, or in combination with elevated temperature, has been implicated as the major factor influencing the structure of viral proteins and virus infectivity. Adenovirus 2 (Ad2) underwent a structural transition at elevated temperatures, accompanied by the loss of penton bases and their fibres at the icosahederal vertices, which are required for the maintenance of viral infectivity [34]. While this structural change caused Ad2 aggregation, the structure change, not the aggregation, appeared to be the direct cause of virus inactivation (Figure 3). This temperature-induced structure change was greater at increasing pH. 

Similarly, Ad5 (Adenovirus 5) demonstrated structural change of capsid with increasing temperature, which was enhanced further at higher pH [35]. Using a fluorescence probe, the temperature dependence of fluorescence showed that increasing temperature resulted in initial loosening of the capsid structure and then more complete capsid disassembly, both leading to virus inactivation. Although these capsid structure changes led to virus aggregation, it appears that the structure changes themselves were responsible for inactivation. Since both cases are concerned with non-enveloped viruses, the effects of temperature are primarily on viral proteins. 

Norwalk virus like particles (VLP), a non-enveloped virus, demonstrates a unique dependence on pH and temperature stresses. When the virus preparation was brought to pH 8.0, intact VLP virus disappeared, most likely due to changes in the local conformational changes of the viral proteins [36]. When this preparation was brought back to pH 7.0, fully assembled infectious virus was recovered: *i.e.*, pH-induced structure change is reversible. However, response to high temperature stress is different. Both the local structural changes of the proteins and the capsid structure upon heating were irreversible. It appears that this irreversible heat-induced change is due to aggregation of viral proteins. 

Another stress that causes virus inactivation is dehydration, which can occur both *in vitro* and *in vivo*. Measles virus is unstable in the dried state at elevated temperatures [40]. Although spray drying procedure causes little changes in its viral infectivity, virus was rapidly inactivated during storage at 37 ºC [41]. Since drying itself had no effect on virus infectivity, there must be a stress or several stresses in the storage condition responsible for time-dependent virus inactivation. Arginine at 0.23 M in pre-drying concentration, insufficient by itself to cause virus inactivation, resulted in the protection of the virus during storage in the dried state [41]. In this case, arginine did not demonstrate synergy with stress (*i.e.*, dehydration) to cause virus inactivation.

### 3.2. Mechanism-2

Aggregation of virus is a common observation [42], although its relation to inactivation may depend on how the virus samples are prepared (see Figure 4). Virus aggregation can now be more readily and accurately characterized using modern technologies, including AUC (analytical ultracentrifugation), DLS (dynamic light scattering) and FFF (field flow fractionation). For example, sedimentation velocity analysis (using AUC) showed that adenovirus aggregates into highly heterogeneous sizes with the monomer population accounting for only ~50% (Alliance Protein Laboratories homepage, www.ap-lab.com).

Aggregation of infectious poliovirus has been extensively studied [37]. A monoclonal antibody, MoAb 35-1f4, was shown to cause polymerization of poliovirus, presumably through its effects on surface proteins, *i.e.*, bivalent binding and/or consequent conformational changes. This polymerization resulted in loss of infectivity. As the size of oligomers increased, infectivity was reduced (e.g. the dimeric virus has 50% reduced infectivity), suggesting that the loss of infectivity was due to steric hindrance of virus accessibility to the host cells and not due to irreversible damages that the virus has suffered from the bound antibody. This was confirmed by the release of infectious virus from the polymerized structure through papain treatment. Polymerization of poliovirus was at least in part due to bivalent antibody binding, as excess antibody resulted in monomeric virion particles: this is expected from a large excess of antibody per binding site on viral particle [43]. Aggregation of infectious viruses is problematic for inactivation. Aggregated viruses are often non-neutralizable by antiserum and cause persistent and delayed infectivity [44]. In this case, viruses may have to be disaggregated for efficient virus inactivation, as will be discussed later.

Structural damages on viruses are often accompanied by virus aggregation. In such cases, virus inactivation and aggregation are associated with each other and hence it is difficult to distinguish between damage-induced inactivation and aggregation-induced inactivation, as illustrated in the following examples. Pinto *et al.* have used DLS to study the aggregation behavior of MS2 bacteriophage, which ranged in size up to 500 nm, and have correlated aggregation to its reduced efficacy [45]. DLS was also employed to correlate the aggregation of influenza virus particles to loss in antigen activity [46]. Furthermore, a 2-log decrease in infectivity of baculovirus was reported to occur concurrently with virus aggregation, upon incubation above 45 ºC [47]. The size of the virus particle was shown to increase from 150 to 250 nm by DLS measurements. Both low pH and high temperature have been shown to be the major factors responsible for virus inactivation associated with aggregation. Measles virus (MV) was shown to aggregate by DLS measurements immediately upon lowering the solution pH from 6 to 4 [32]. With increasing temperature, both the aggregate size and measurement variability of MV increased further. The cause of aggregation is still unknown, however membrane destabilization due to the instability of the membrane proteins, and thus the lipid-protein interaction, may help to explain the observation. Thermally-induced changes in the secondary structure of MV viral proteins was detected using circular dichroism (CD) at 222nm as a function of temperature, and the temperature at which this transition occurred was decreased by more than 30 ºC upon lowering the pH from 6 to 4. Namely, low pH may cause the conformational changes of MV viral proteins at physiological temperatures, which in turn affect membrane structure of MV and lead to virus aggregation. 

In another example of low pH-mediated aggregation, respiratory syncytial virus (RSV) underwent extensive aggregation under acidic conditions; aggregation of RSV at pH 3–5 during dialysis was confirmed by elevated optical density values [33]. Further evidence of membrane destabilization leading to aggregation was obtained from the intrinsic fluorescence data; the temperature at which the changes in the tertiary structure of viral proteins was detected correlated with the temperature at which increases in turbidity occurred, which suggest that the exposure of the apolar tryptophan (Trp) residues led to aggregation of RSV particles. CD analysis revealed that the temperature (T_d_) at which the RSV membrane proteins lost secondary structural content varied depending on the solution pH; significant loss was observed below 40 ºC at pH 3–5 and in between 40–60 ºC for solution pH between 6 and 8. FTIR (Fourier transform infrared spectroscopy) analysis indicated that the cause of this shift in T_d_ was primarily due to the formation of intermolecular β-sheets, which may be due to aggregation. Thus, there is an abundance of data implicating the structural instability of viral proteins to be the main cause for virus aggregation, which consequently leads to its inactivation. It is still unclear, however, why the viral membrane is unstable under acidic condition and not under alkaline condition. 

### 3.3. Mechanism-3

Freezing causes loss of infectivity of enveloped viruses [48], suggesting that ice crystal formation damages membrane lipids, as depicted in Figure 5 [49,50]. Consistent with this notion, DMSO (dimethyl sulfoxide), which is commonly used to preserve cultured cells, protected several viruses form freezing-induced loss of infectivity [51]. 

Defensins, which comprise a family of broad-spectrum antimicrobial peptides, are among the key mediators of the innate immune system. One of the defensin family member, human neutrophil peptide (HNP-1), was shown to be effective in inactivating several viruses, including HSV types 1 and 2, cytomegalovirus, vesicular stomatitis virus, and influenza virus A/WSN [38]. Similarly to arginine, an enhancement in HNP-1’s virucidal efficacy was observed upon modifying either the solution pH or the temperature; increasing the incubation temperature from 10 to 40 ºC resulted in >3Log_10_ reduction in infectivity of HSV-1 in the presence of 25 μg/mL HNP-1 at pH 7.4, while increasing the pH from 6 to 8 in the presence of HNP-1, reduced the infectivity by >1 Log_10_ at 37 ºC. Defensins are not effective against all viruses, however. HNP-1 was not effective against echovirus and reovirus, which are both non-enveloped viruses. Similarly, arginine was shown to be ineffective against poliovirus, a non-enveloped virus [10]. The difference in susceptibility observed between enveloped and non-enveloped viruses suggests that the viral envelope may be the target of both defensins and arginine, as described later. The correlation between reduction in infectivity and the interaction of these classes of compounds to the viral envelope suggests that the interaction leads to a breach in the integrity of the viral envelope. Evidence to support this statement can be found in a study that was conducted to determine the effect of human defensin HNP-2 on the integrity of unilamellar vesicles [39]. A fluorescence quenching study, utilizing ANTS-DPX (8-aminonaphthalene-1,3,6 trisulfonic acid - *p*-xylene-bis-pyridinium bromide) system encapsulated within a POPG (palmitoyloleoylphosphatidylglycerol) vesicle, was conducted to observe the effect of increasing HNP-2 concentration on the integrity of the liposome. The addition of 1.3 μM HNP-2 to POPG vesicles resulted in enhanced fluorescence, which further increased with incubation time. Decreasing the HNP-2 concentration from 1.3 to 0.3 μM resulted in a decrease in both the fluorescence signal and the release kinetics, suggesting that the interaction is concentration dependent. The similarity observed between arginine and defensins may not be unexpected, as mature neutrophil defensins are reported to comprise of high arginine content [39]. 

## 4. Mechanism of the Virus Inactivation Effects of Arginine

As the surface of enveloped viruses is composed of spike proteins and membrane lipids, these are the most likely targets of arginine. However, any preparations of viruses used for *in vitro* and *in vivo* experiments are usually heterogeneous, containing cell debris, aggregated particles, and chemically or physically modified viruses. Thus, the observed virus inactivation effects of arginine can be complicated by the heterogenous nature of virus preparation.

It appears that pH and temperature are the major cause of alteration in the structure of spike proteins, leading to virus inactivation often accompanied by aggregation. Does arginine further enhance structural changes in viral proteins (See Figure 3, Mechanism-1)? Arginine has been shown to have little impact on the native structure of globular proteins under physiological conditions [16,17], consistent with its inability to inactivate viruses at neutral pH and room temperature. Would then the synergistic effects of arginine be due to its ability to enhance the pH or temperature-induced conformational changes of the viral proteins? Arginine has been shown to have small effects on the melting temperature of globular proteins as summarized in Table 2 [14,16]. Even at 1-2 M arginine, the observed decrease in melting temperature was at most 3 ºC, much smaller than the observed decrease in virus inactivation temperature upon arginine addition (Table 1). Arginine also does not significantly affect the pH-induced conformational changes of antibodies (unpublished information): note that antibodies also consist of globular immunoglobulin domains. It is possible that arginine may have qualitatively different effects on viral proteins. 

**Table 2:** Change in melting temperature.

Arginine Concentration / M	Lysozyme/°C	RNase/°C
0.1	0	0
0.2		-1
0.5	-1	-1
1.0	-1	-3
2.0	0	-3

Melting temperature was determined from the temperature dependent absorbance changes as described in the original manuscript [14]. Melting temperature in the absence of arginine was subtracted from that in the test solvents. RNase, ribonuclease. Reformatted from reference [14].

Arginine suppresses protein-protein interactions and aggregation [15,16,17]. If pH- or temperature-induced virus inactivation involves the alteration of protein-protein interactions, it is possible that the suppressive effects of arginine on the interactions may augment virus inactivation (see Figure 5). As stated above, these two stresses (pH and temperature) often lead to virus aggregation, which may be the protective mechanism employed by the viruses to shield themselves from further damages by the stresses encountered [43,44]. As arginine has been shown to suppress virus aggregation, its addition may enhance the damages caused by pH or temperature stress on the disaggregated virus particles. 

Although unrelated to virus aggregation, arginine can also inhibit agglutination processes. Certain viruses induce agglutination of erythrocytes, and the effects of solvents on agglutination may potentially mimic virus inactivation processes. Namely, the solvents that alter viral protein structure, and consequently reduce agglutination, may cause virus inactivation. Measles, EMC and NDV viruses associate with erythrocyte cell membrane and induce hamagglutination [52]. These interactions are readily dissociated by 0.1–1 M arginine at pH 7.0, leading to the recovery of virus infectivity. Furthermore, this condition is insufficient for virus inactivation. Nevertheless, hemagglutination may be used to screen solvents for their ability to alter the structure of viral proteins.

Aggregated viruses may respond to pH and temperature differently. They are persistent to virus inactivation [43,44]. As schematically depicted in Figure 4, arginine may dissociate viruses, which on one hand may increase the number of infectious viral particles, while on the other hand, may make the virus more susceptible to pH or temperature stress. The effects of arginine on aggregated infectious viruses may be determined by the fine balance between dissociation-induced virus activation and stress-induced damages on dissociated viruses.

Arginine has also been shown to suppress the interactions of proteins with non-polar surfaces [24,53,54]. Such effects may have an impact on the protein-lipid interface of virus particles. As described above, pH- and temperature-induced changes in viral proteins affect membrane stability. Arginine can affect both protein-lipid and lipid-lipid interactions. In fact, arginine has been shown to suppress the formation of oil droplets [20], and peptides containing multiple arginines, as in defensins and twin-arginine transporters, have been reported to penetrate membranes [55,56,57]. Arginine-containing peptides have been demonstrated to bind to the surface of the lipid membrane and create transient pores across the bilayer. Small arginine peptides can be inserted into the membrane while large peptides could be efficiently internalized. Although the precise mechanism of the interaction between arginine and lipid is not clear, arginine has been proposed to bind to phosphate groups [58,59]. This binding does not require charged arginine to move into non-polar environment of low dielectric constant. However, penetration of arginine peptides requires the transfer of arginine side chain to low dielectric media of membrane, and hence, high energy interaction. Recently, arginine residues have been shown to be incorporated into lipidic environments as charged species, which for unknown reason is not energetically unfavorable [60,61]. This does, however, cause perturbation of lipid-lipid interactions. Both effects of arginine, *i.e.*, binding to phosphate and insertion into lipid membrane, may be responsible for the virus inactivation observed upon the addition of arginine, as depicted in Figure 5 (Mechanism-3). 

The effects of arginine are due to weak interactions with virion components, independent of the inactivation mechanisms. This is evident from the requirement of high concentrations of arginine. Such weak, but extensive interactions of arginine and many other compounds have been shown to occur with a variety of proteins and macromolecules [62,63,64,65,66]. NaCl is one example and demonstrated dual effects on proteins, *i.e.*, electrostatic ionic binding and weak salting-out effects [64]. The observed synergistic effect of NaCl with elevated temperature on influenza virus inactivation (Table 1) may be due to its ionic property. This may also explain the effect observed with ionic arginine. However, virus inactivation by NaCl may be due to its salting-out effect [64]. In such a case, NaCl and arginine, which exhibit salting-in effects, must have an entirely different mechanism for influenza virus inactivation. In other words, influenza virus can be inactivated by both salting-out mechanism of NaCl (for example, due to virus aggregation) and salting-in mechanisms of arginine (suppression of interactions). Such weak interactions are also involved in stabilization of viruses. For example, 1 M NaCl stabilized the Ad5 viruses against heat stress due to its stabilizing effect of the viral proteins. It is interesting to note that the addition of NaCl can both destabilize (*i.e.*, influenza virus) and stabilize (*i.e.*, Ad5) a virus against the same stress, heat, in this case. Similarly, sucrose stabilized RSV [67]. When 20% sucrose was added to the preparation of RSV, heat-induced virus aggregation was inhibited by 66%, which was ascribed to the stabilization of viral glycoproteins. Arginine also stabilized the RSV against heat stress, presumably due to its salting-in effects. It appears that arginine prevents the aggregation of thermally unfolded viral proteins.

**Figure 6:**
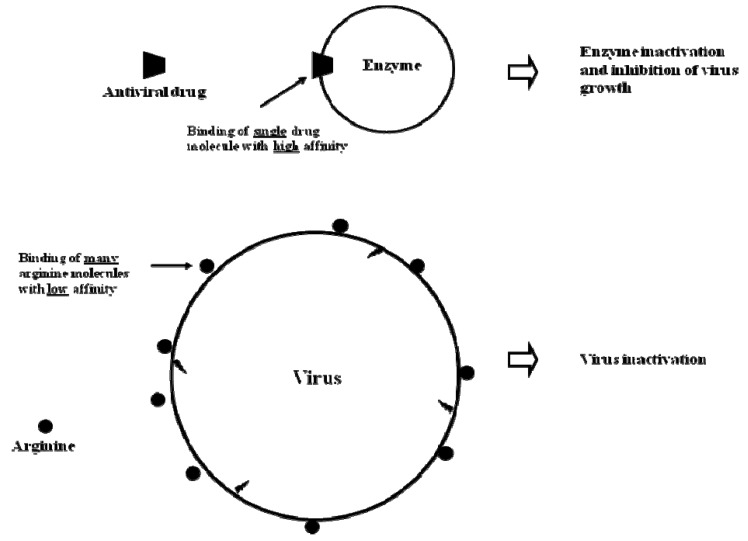
High affinity binding of specific antiviral drug and weak arginine binding.

Weak interactions have two important implications. Binding of arginine is normally reversible. When arginine concentration is reduced, it dissociate from the complexes. The consequence of binding can be, however, irreversible as in the case of virus inactivation. This implies that arginine could be less toxic than those disinfectants that strongly (and irreversibly) bind. Another important implication is that multiple arginine binding at different sites on a virus will unlikely cause drug resistance. It would be a daunting task for the virus to mutate all of the potential targets of arginine (see Figure 6). On the other hand, conventional antiviral drugs strongly bind to the target enzymes and proteins at the specific sites and often lead to drug resistance, as depicted in Figure 6.

## 5. Conclusions

We have shown three possible modes that cause virus inactivation: structural changes of viral spike proteins, virus aggregation and pore formation in virus envelope. Arginine can inactivate viruses synergistically with other stresses, e.g., elevated temperature or acidic pH through any one or combinations of these modes. The mechanism by which arginine exerts its virus inactivation effect is most likely due to the suppression of protein interactions with other molecules or surfaces. More importantly, such effect of arginine is due to its weak interactions, which are unlikely to lead to the development of virus resistance against arginine.
